# Prevalence of Incidental Findings and Assessment of Maxillary Sinus Pathologies and Dental Diseases Using Cone-Beam Computed Tomography (CBCT) in the Tamil Nadu Population: A Retrospective Study

**DOI:** 10.7759/cureus.68929

**Published:** 2024-09-08

**Authors:** Sivaraman GS, Ramasamy Sarvathikari, Ahamed AMHR Alkandari, Sudhamani, M Khaja Khalid Nawaz, Jayasharmila L, Shanmathy Sureshbabu, Abirami Arthanari, Karthikeyan Ramalingam

**Affiliations:** 1 Department of Oral Medicine and Radiology, Vivekanandha Dental College for Women, Namakkal, IND; 2 Department of Oral Medicine and Radiology, Annamalai University, Cuddalore, IND; 3 Department of Dentistry, South Khaitan Health Center, Kuwait, KWT; 4 Department of Oral and Maxillofacial Surgery, Dr. H. Gordon Roberts Hospital, Kuwait, KWT; 5 Department of Forensic Odontology, Saveetha Dental College and Hospitals, Saveetha Institute of Medical and Technical Sciences, Saveetha University, Chennai, IND; 6 Department of Oral Pathology and Microbiology, Saveetha Dental College and Hospitals, Saveetha Institute of Medical and Technical Sciences, Saveetha University, Chennai, IND

**Keywords:** cone beam computed tomography (cbct), dental diseases, diagnostic imaging cognitive-behavioral therapy, incidental findings, maxillary sinus pathologies, oral health, prevalence, radiographic assessment, retrospective study, tamilnadu population

## Abstract

Background

Cone-beam computed tomography (CBCT), a cross-sectional imaging technique, is valuable for clinical diagnosis and creating effective treatment plans. CBCT can precisely examine the connection between the maxillary sinuses and the maxillary root apices. Oral radiologists must be aware of all potential incidental findings and should be diligent in thoroughly identifying and assessing possible underlying diseases.

Aim

To determine the prevalence of incidental maxillary sinus pathologies and their relationship to dental diseases.

Materials and methods

In the present retrospective study, CBCT scans from 300 subjects (encompassing 600 right and left maxillary sinuses), aged 18 to 70, were gathered from various CBCT centers to represent the Tamil Nadu population. The CBCT images were analyzed using proprietary software, which provided both a panoramic reconstruction view and multiplanar reformation modules, including axial, sagittal, and coronal slices. The entire sample size was classified as follows: Group 1, age groups of 18 to 25 years; Group 2, age groups of 26 to 35 years; Group 3, age groups of 36 to 45 years; Group 4, age groups of 46 to 55 years; Group 5, age groups of 56 to 65 years; Group 6, age groups of 66 to 70 years. The prevalence of incidental maxillary sinus findings was analyzed, and their relationship with periapical abscess, periapical granuloma, periapical cyst, and breach was assessed.

Results

There was a prevalence of 52.05% of cases that had incidental maxillary sinus findings. Among them, 53.43% were males and 50.65% were females. Maxillary sinus pathologies were more common in individuals between 46 and 55 years, i.e., Group 4. In 300 datasets, the frequency of incidental maxillary sinus findings on the right is 21.33%, on the left is 24%, in both is 6.67%, and absent in 48% of the cases. Mucosal thickening was observed in 30% of cases with a periapical abscess, 19.52% with a periapical granuloma, 25% with a periapical cyst, and 51.79% with a breach. Polypoidal mucosal thickening was present in 32.50% of cases with a periapical abscess, 13.79% with a periapical granuloma, 50% with a periapical cyst, and 23.21% with a breach. Opacification occurred in 37.50% of cases with a periapical abscess, 20.69% with a periapical granuloma, 25% with a periapical cyst, and 25% with a breach.

Conclusion

Dental professionals should have a bird's-eye view in treating chronic odontogenic infections close to the maxillary sinus. Early diagnosis and prompt treatment of odontogenic infections help prevent maxillary sinus pathologies.

## Introduction

Introduced in 1998, cone-beam computed tomography (CBCT) is increasingly utilized for three-dimensional imaging in maxillofacial radiology, serving as an alternative to traditional fan-beam helical computed tomography (CT) machines. CBCT allows for faster acquisition of the entire field of view's dataset, reducing the need for patient movement and decreasing scan time [1]. Unlike other extraoral radiographs, CBCT offers vital information on both dental and maxillofacial structures. Compared to conventional CT, CBCT is more efficient in this regard [2,3]. CBCT offers lower radiation doses to patients compared to conventional CT machines, utilizing an image intensifier. Additionally, CBCT machines are significantly less expensive than traditional CT machines. This cross-sectional imaging technique is valuable for clinical diagnosis and the formulation of effective treatment plans. Common applications include assessing dental implant sites, orthodontics, treating maxillofacial trauma and infections, managing dental endodontic issues, evaluating temporomandibular joint (TMJ) bone conditions, assessing bone quality and quantity for dental implants in edentulous patients, addressing developmental and congenital jaw abnormalities, managing impacted and supernumerary teeth, and diagnosing oral and maxillofacial diseases [4].

On the other hand, the advantages of CBCT are high contrast and high image quality of structures, no superimposition of surrounding anatomical structures, and no geometric distortion. CBCT also plays a major role in predicting oroantral communications before tooth extraction [5].

The increase in the use of CBCT creates opportunities for dentists and maxillofacial surgeons to assess the prevalence of incidental findings of the maxillary sinus, facilitating more thorough evaluations. It allows for the volumetric assessment of investigated structures using different slices and avoids the need for multiple 2D images. Identifying the relationship between the sinus and odontogenic pathologies is essential for establishing the correct diagnosis and management of the patient [6].

To prevent unnecessary treatments or to ensure appropriate treatment and follow-up, it is important to understand the significance of incidental pathological findings in CBCT scans. This study aimed to retrospectively identify the location, nature, and frequency of incidental findings observed in maxillofacial CBCT scans conducted for diagnostic purposes in maxillofacial contexts [7].

## Materials and methods

Source of data

The current study is retrospective and was carried out in the Department of Oral Medicine and Radiology.

Ethical clearance

A detailed protocol outlining the aim and methodology of the study was submitted to, and approved by, the Institutional Ethical Committee (VDCW/IEC/314/2022) before the study commenced.

Sample size

CBCT scans from 300 subjects (comprising 600 right and left maxillary sinuses) were gathered from multiple CBCT centers located in Madurai, Chennai, Salem, and Tirunelveli, aiming to reflect the demographic diversity of Tamil Nadu. The study details were communicated to the CBCT centers, and consent was obtained from the participants. The selection of CBCT images adhered to specific inclusion and exclusion criteria.

Inclusion criteria

The study involved subjects who were in the age range of 18-70 years and those who belonged to the Tamil Nadu population. The primary reasons for CBCT were endodontic procedures, TMJ evaluations, trauma involving the maxilla, and periodontal pathology.

Exclusion criteria

Exclusion criteria include patients under 18 years of age, patients with maxillofacial trauma affecting the middle third of the face, or those who have undergone maxillofacial surgery, individuals with bone diseases such as skeletal asymmetries, congenital disorders, or syndromic conditions, and images affected by artifacts.

The entire sample size was classified as follows: Group 1: age groups of 18 to 25 years; Group 2: age groups of 26 to 35 years; Group 3: age groups of 36 to 45 years; Group 4: age groups of 46 to 55 years; Group 5: age groups of 56 to 65 years; and Group 6: age groups of 66 to 70 years. The CBCT images were analyzed using proprietary software that provides panoramic reconstruction and multiplanar reformations, including axial, sagittal, and coronal views. All assessments were conducted under standardized conditions in the same examination setting. Observers were allowed to adjust brightness, contrast, and zoom levels as needed. Calibration for each finding was established beforehand. To minimize bias, two observers independently interpreted the radiographs with a 15-day interval between sessions. Any discrepancies were resolved through discussion and mutual agreement. Following data collection, entries were made into an Excel spreadsheet for analysis, version 7 (Microsoft® Corp., Redmond, WA, USA). The technical parameters used in this study are mentioned in Table 1.

**Table 1:** Technical parameters of the CBCT machine used in this study CCD: Charge-coupled device; FOV: Field of view; CBCT: Cone-beam computed tomography

Parameter	Value
Detector	Image intensifier/CCD
X-ray source voltage	80-90 kVp
X-ray source current	5 mA
FOV	5 x 5 mm to 17 x 13.5 mm
Voxel size	90-500 μm
Slice interval and thickness	0.2 mm

All available views within the software were accessible to the user for the detection of pathological findings, including incidental pathologic maxillary sinus findings.

Mucosal thickening

**Figure 1:**
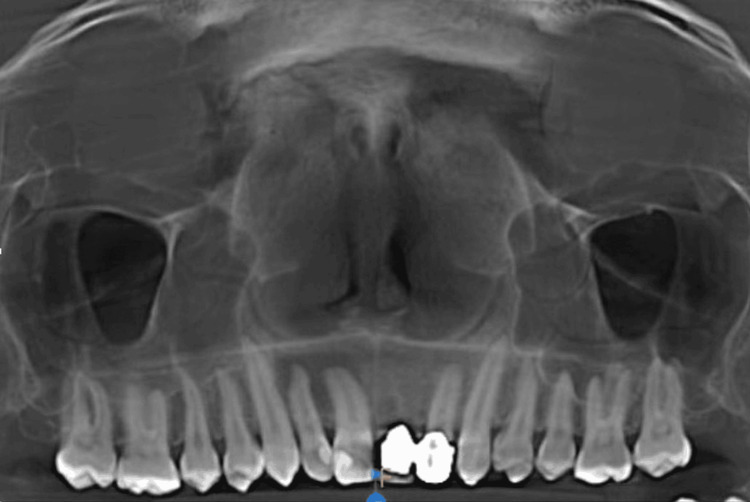
Mucosal thickening - OPG view OPG: Orthopantomogram

**Figure 2:**
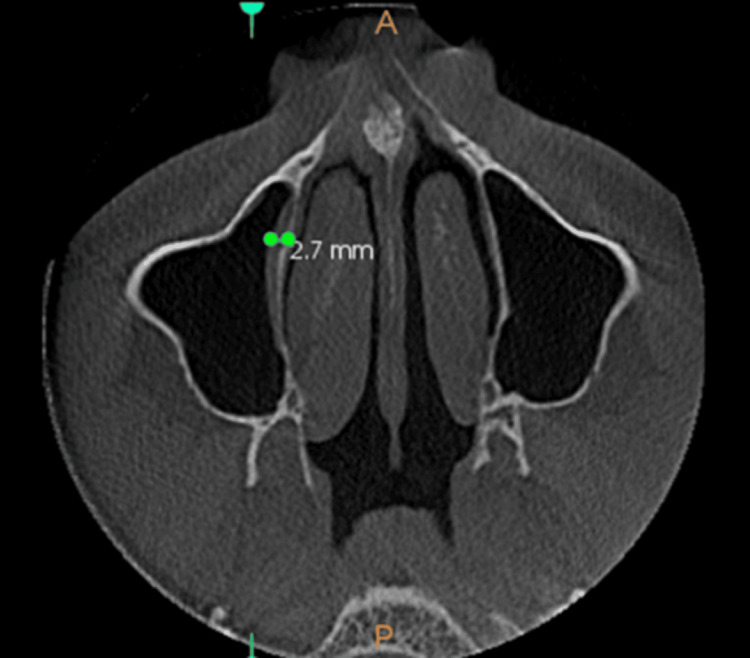
Mucosal thickening - axial view

**Figure 3:**
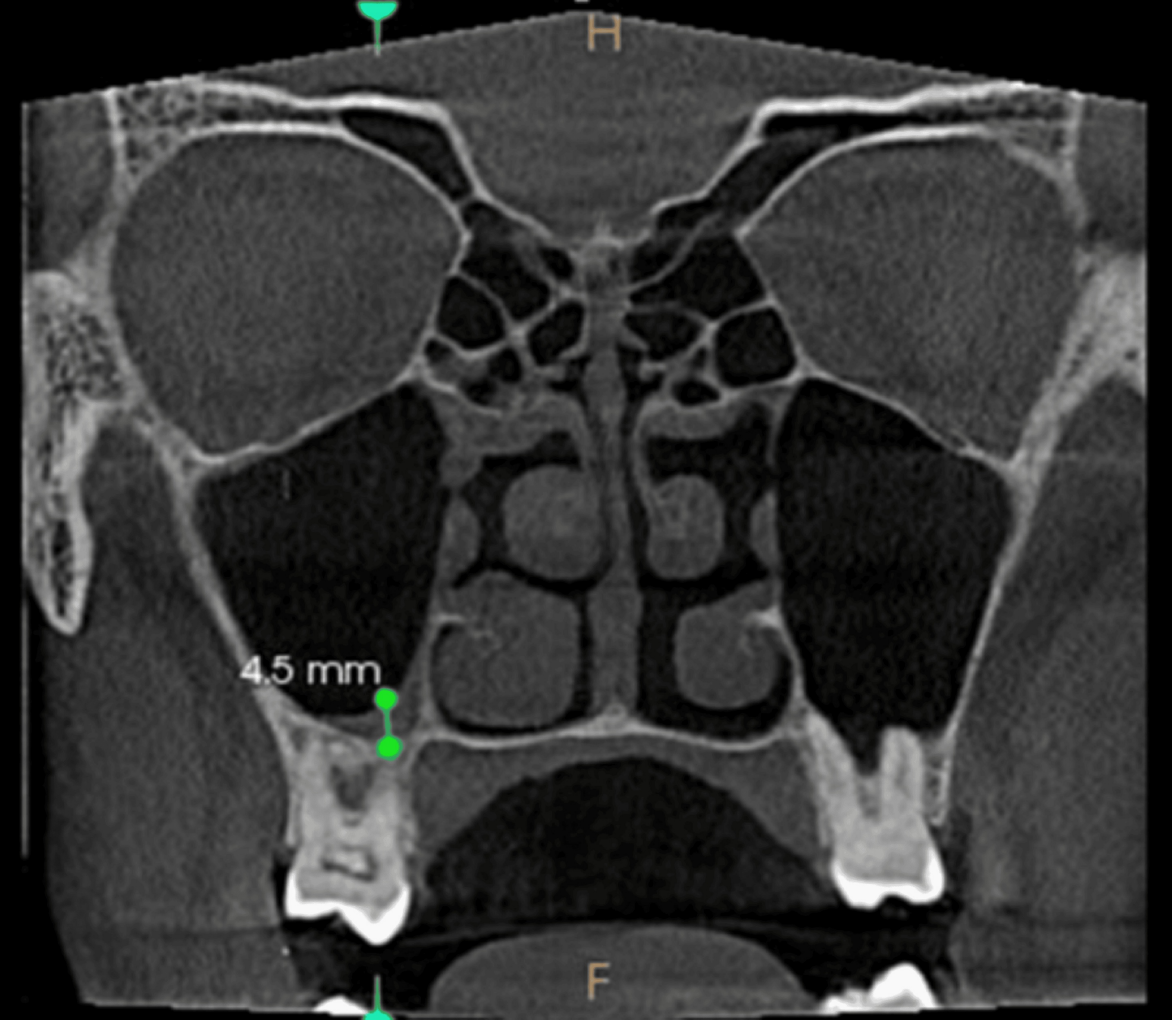
Mucosal thickening - coronal view

**Figure 4:**
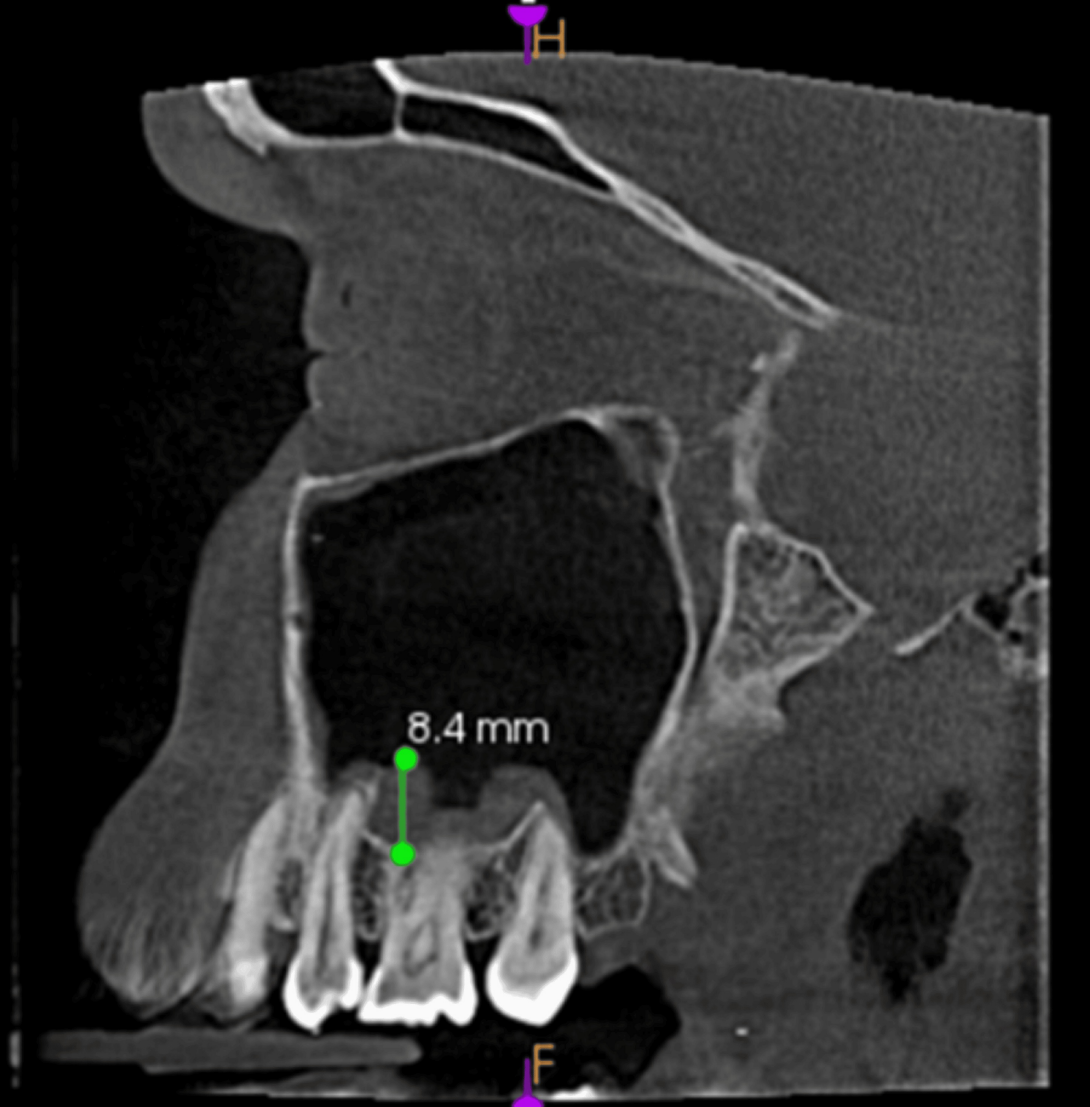
Mucosal thickening - sagittal view

The mucosal thickening in the images taken for crown placement in teeth 21 and 22, in OPG view, axial view, coronal view, and sagittal view, is shown in Figures 1-4.

Polypoidal mucosal thickening

**Figure 5:**
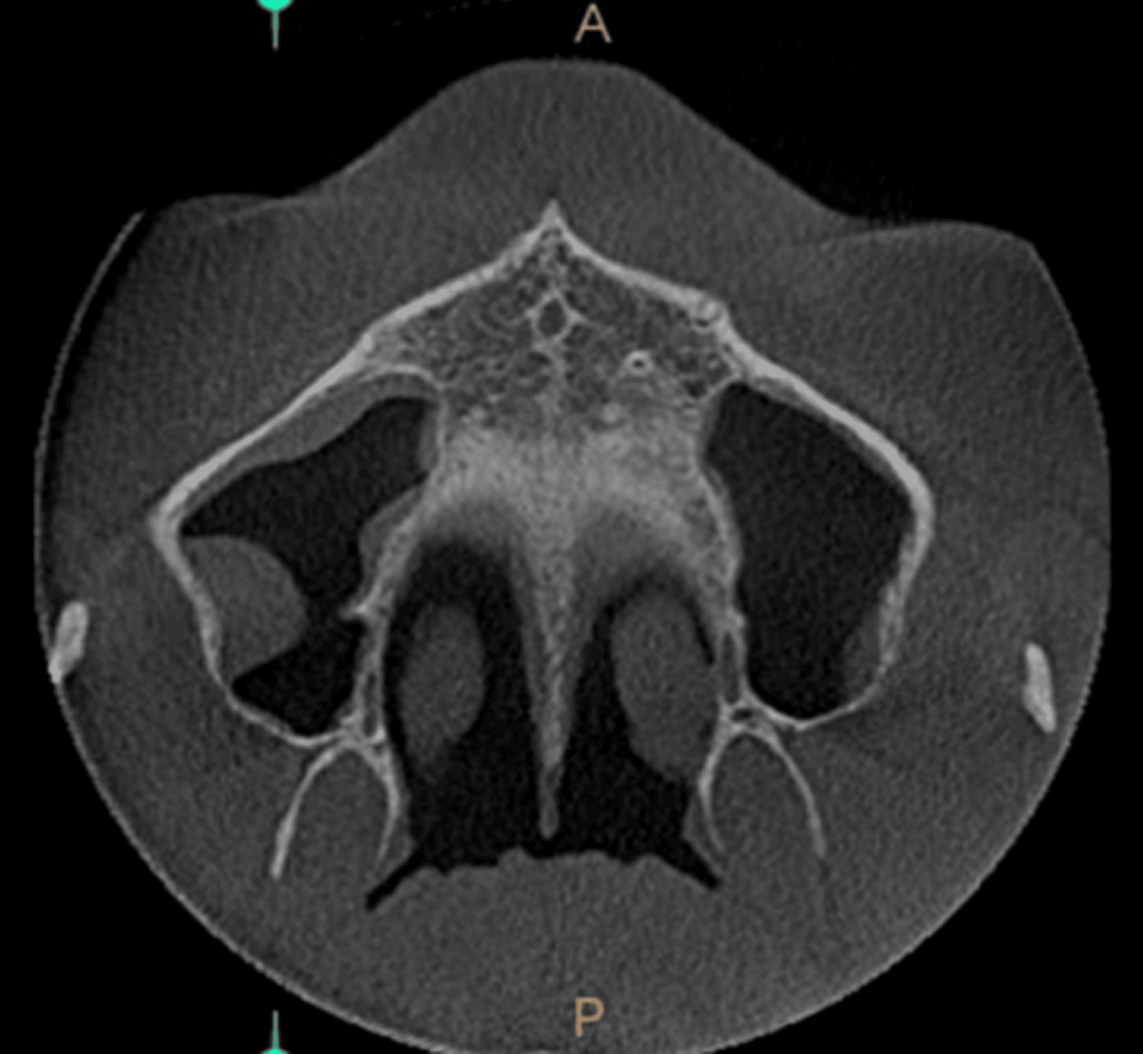
Polypoidal mucosal thickening - axial view

**Figure 6:**
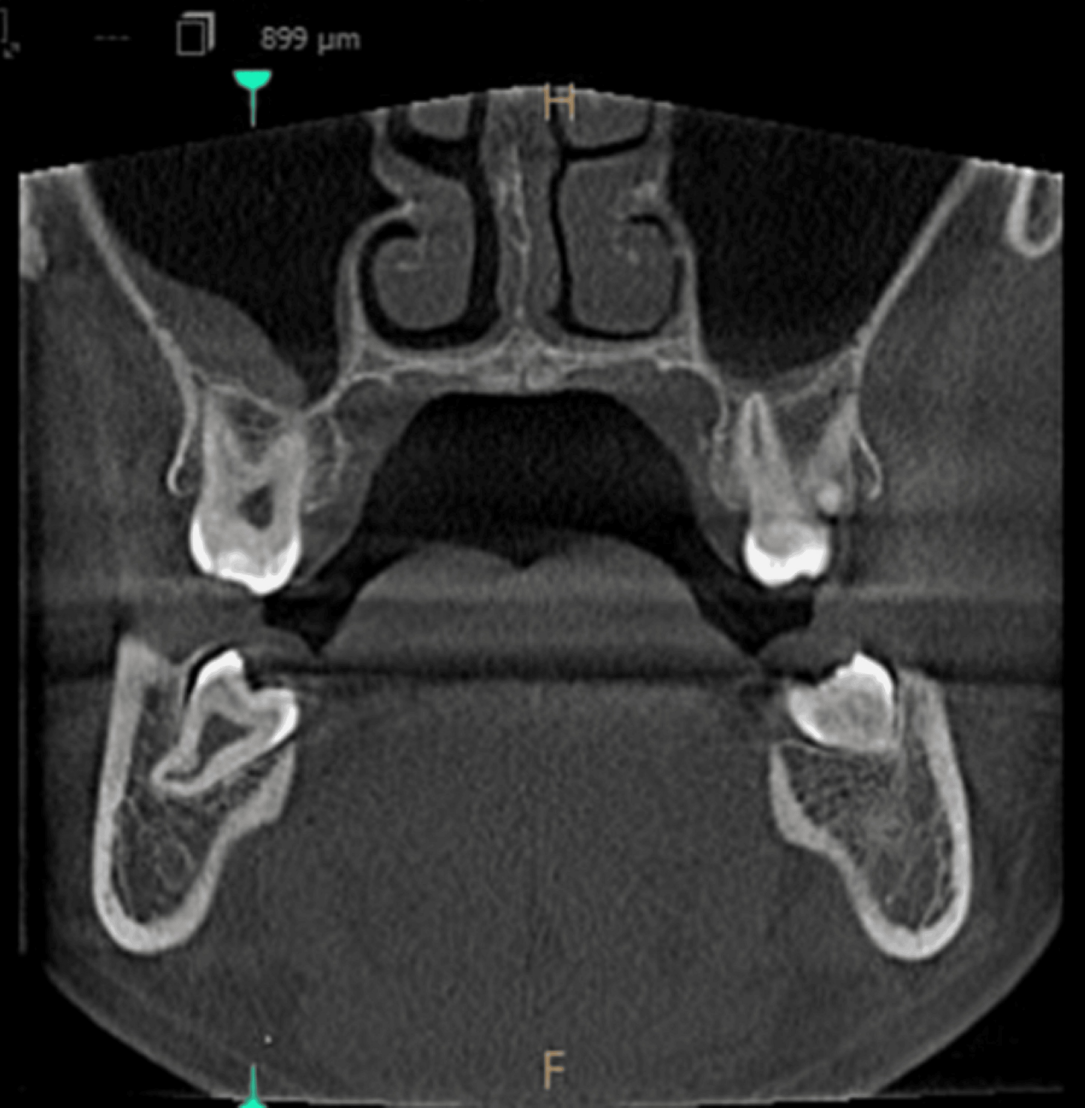
Polypoidal mucosal thickening - coronal view

**Figure 7:**
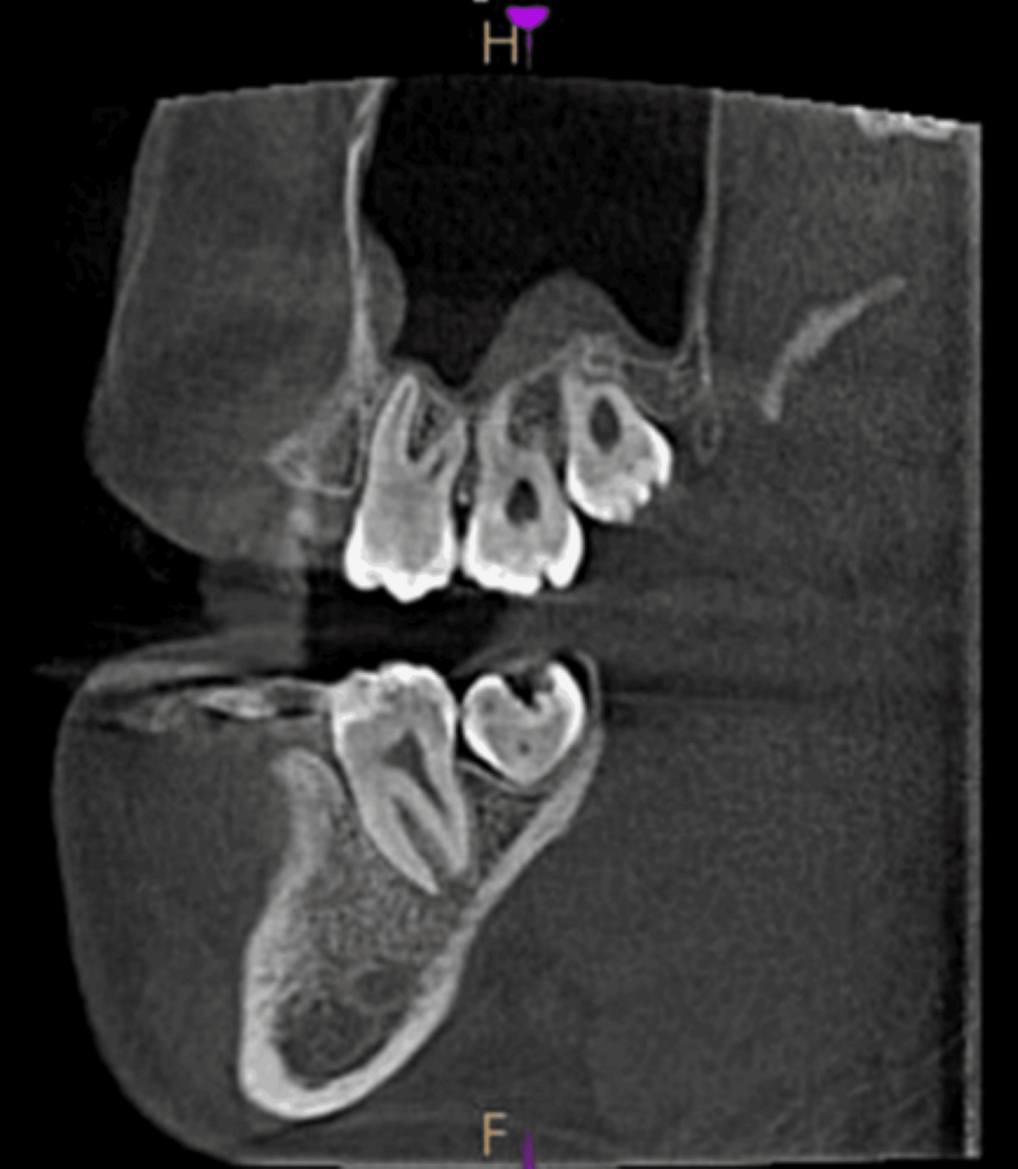
Polypoidal mucosal thickening - sagittal view

Images taken for endodontically treated teeth 45 and 46, in axial view, coronal view, and sagittal view, are shown in Figures 5-7.

Opacification of maxillary sinus

**Figure 8:**
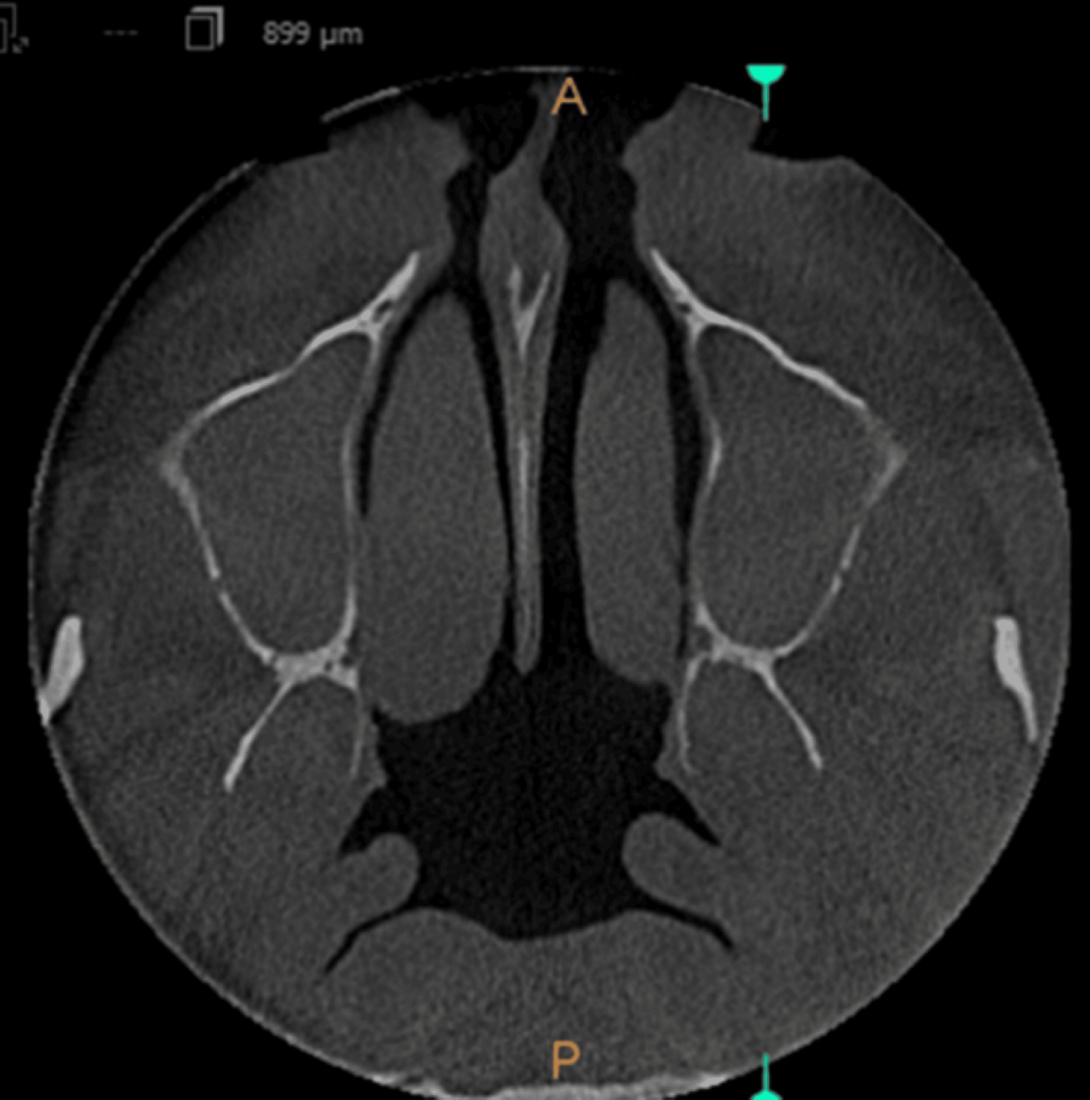
Opacification of the maxillary sinus - axial view

**Figure 9:**
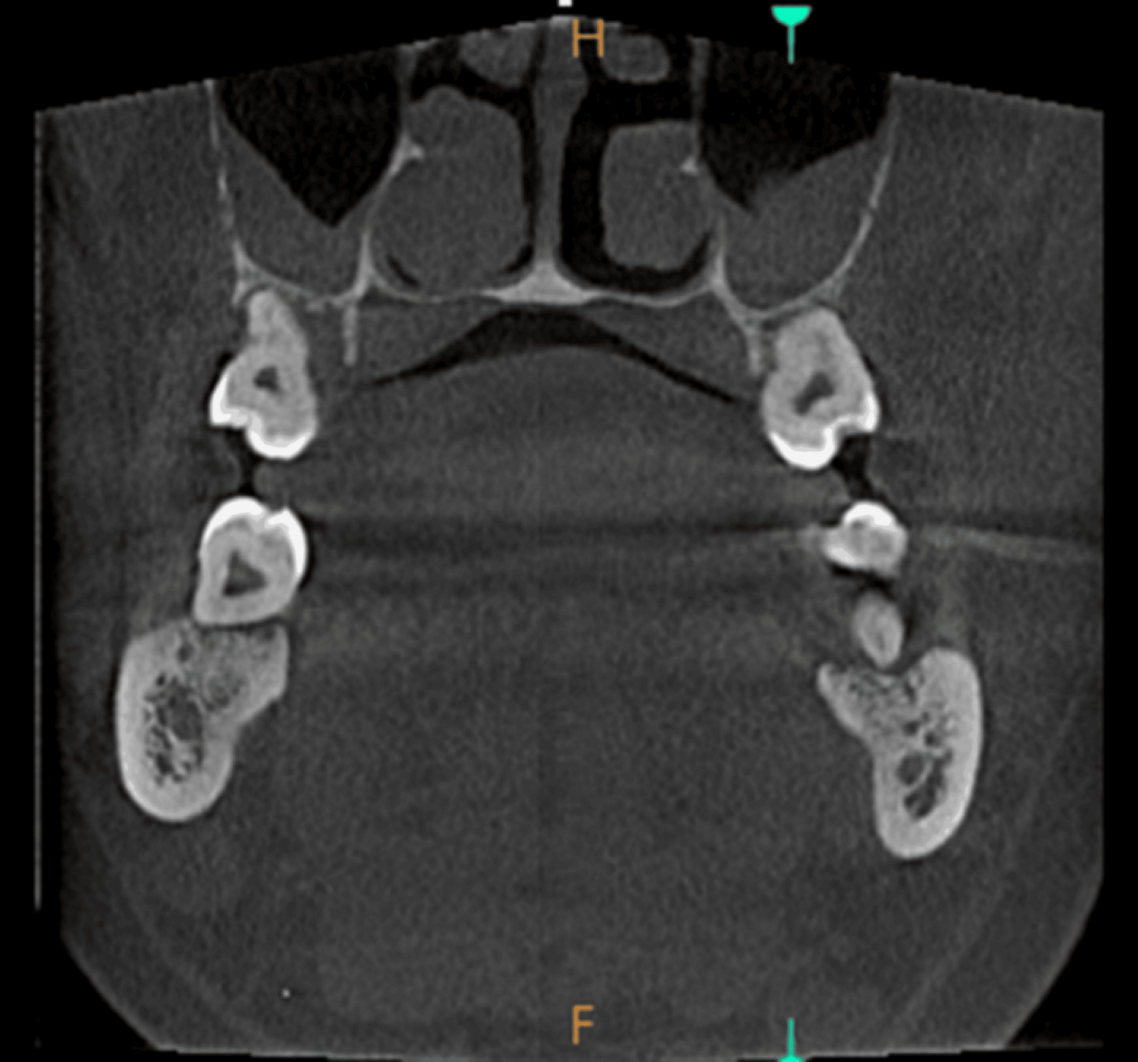
Opacification of the maxillary sinus - coronal view

**Figure 10:**
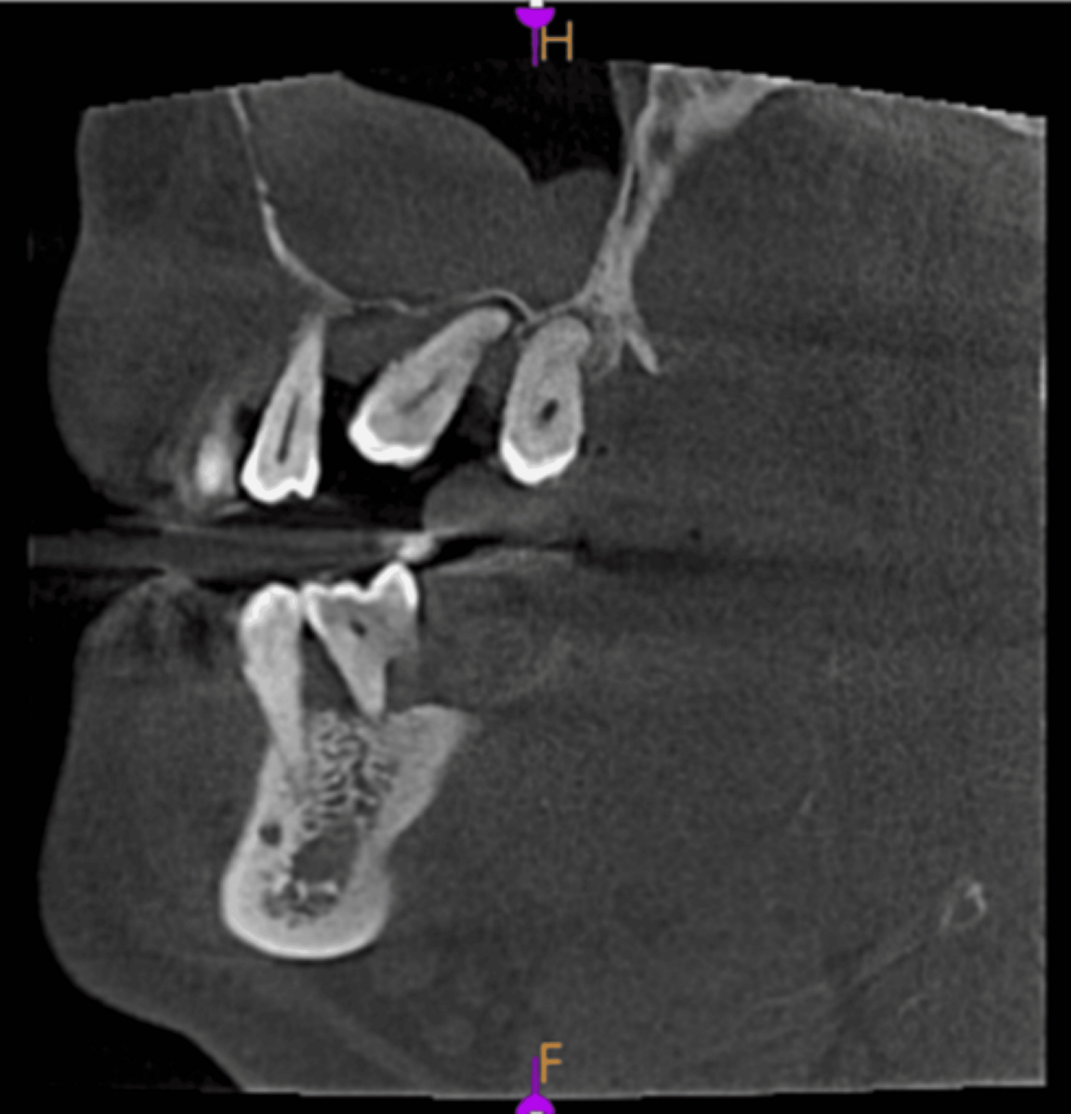
Opacification of the maxillary sinus - sagittal view

Images taken for endodontically treated teeth 32, 41, and 42, in the axial view, coronal view, and sagittal view, are shown in Figures 8-10.

**Figure 11:**
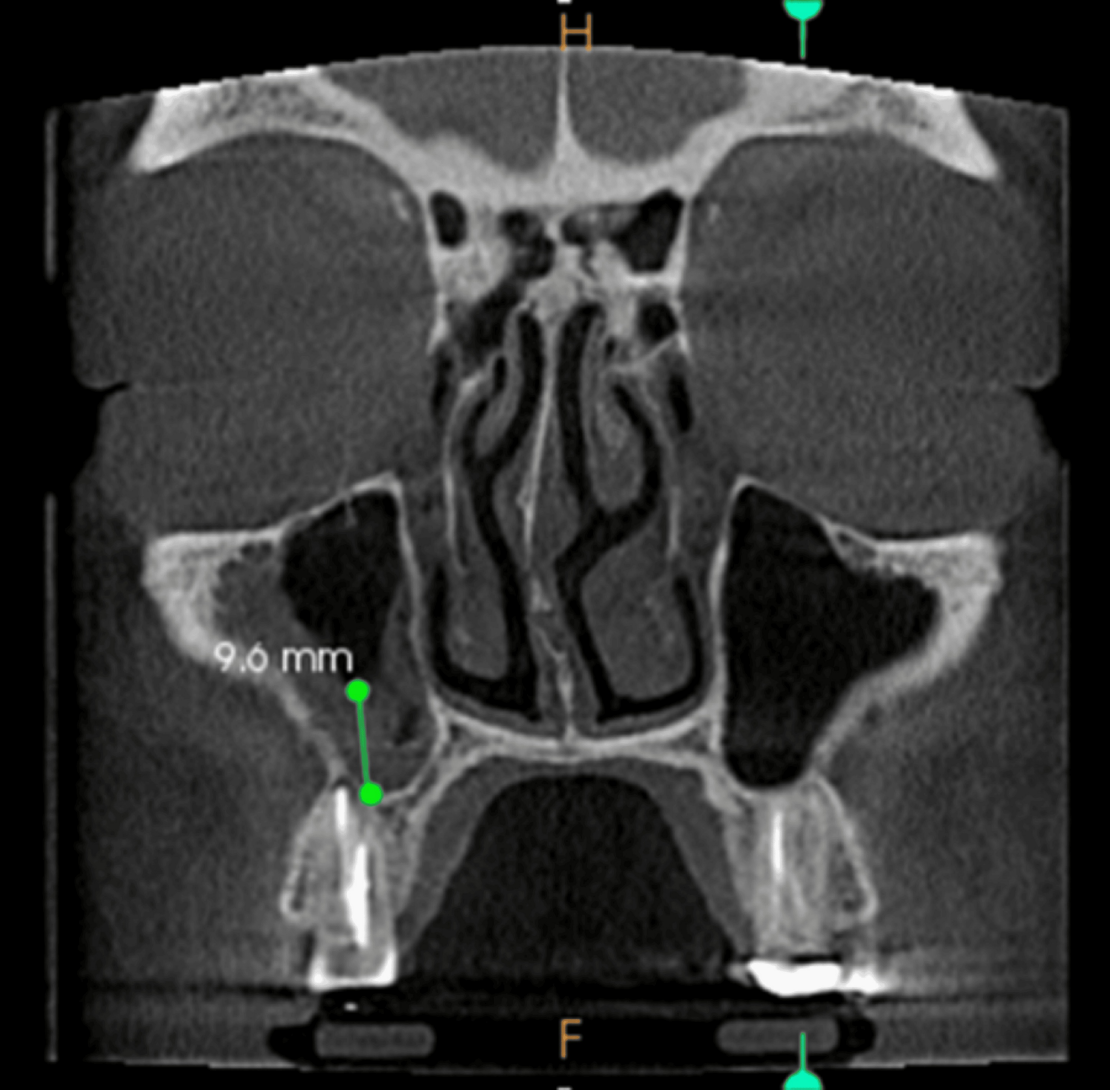
Periapical abscess in 15 with right maxillary sinus mucosal thickening

**Figure 12:**
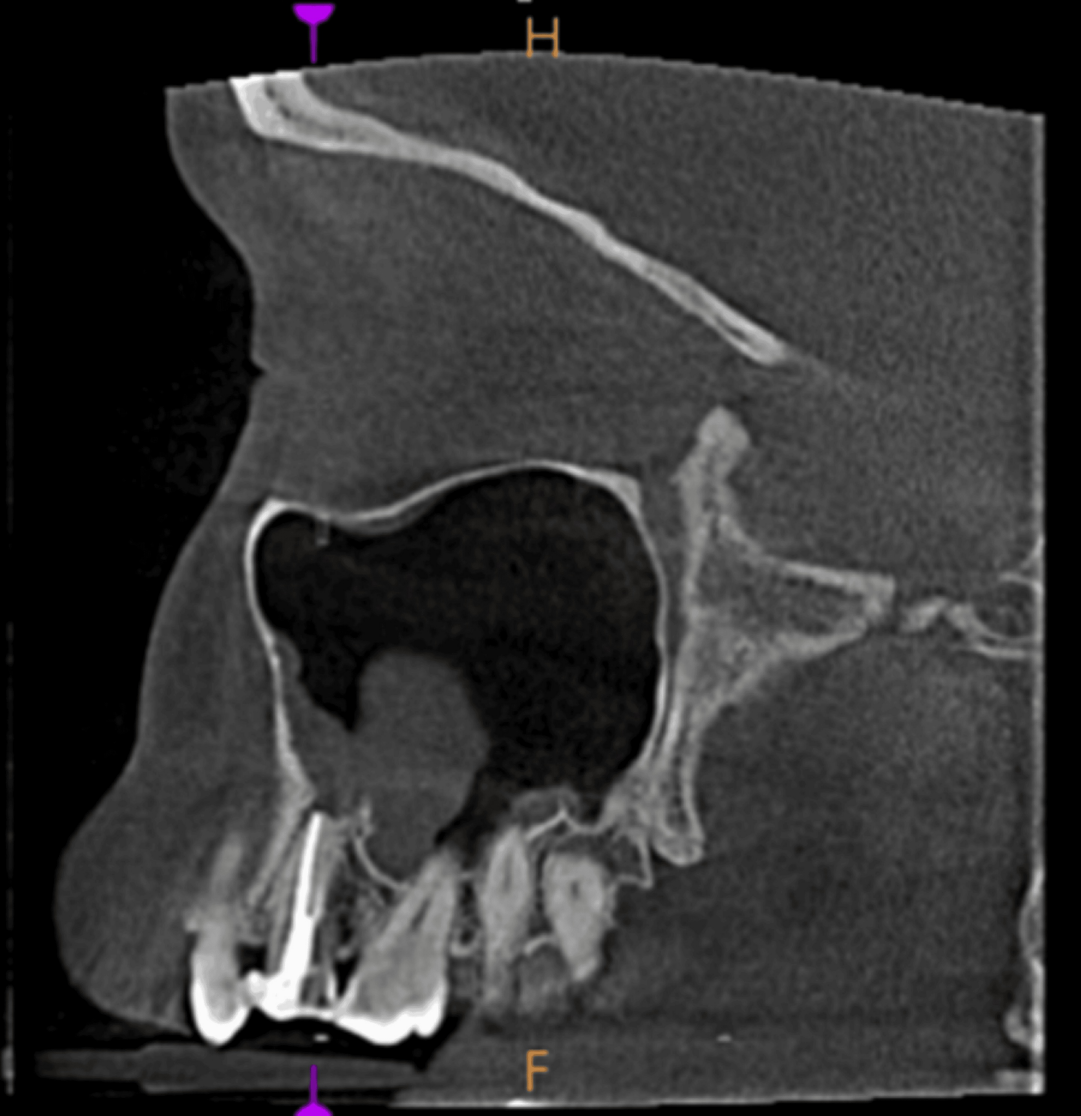
Periapical abscess in 15 with right maxillary sinus polypoidal thickening

**Figure 13:**
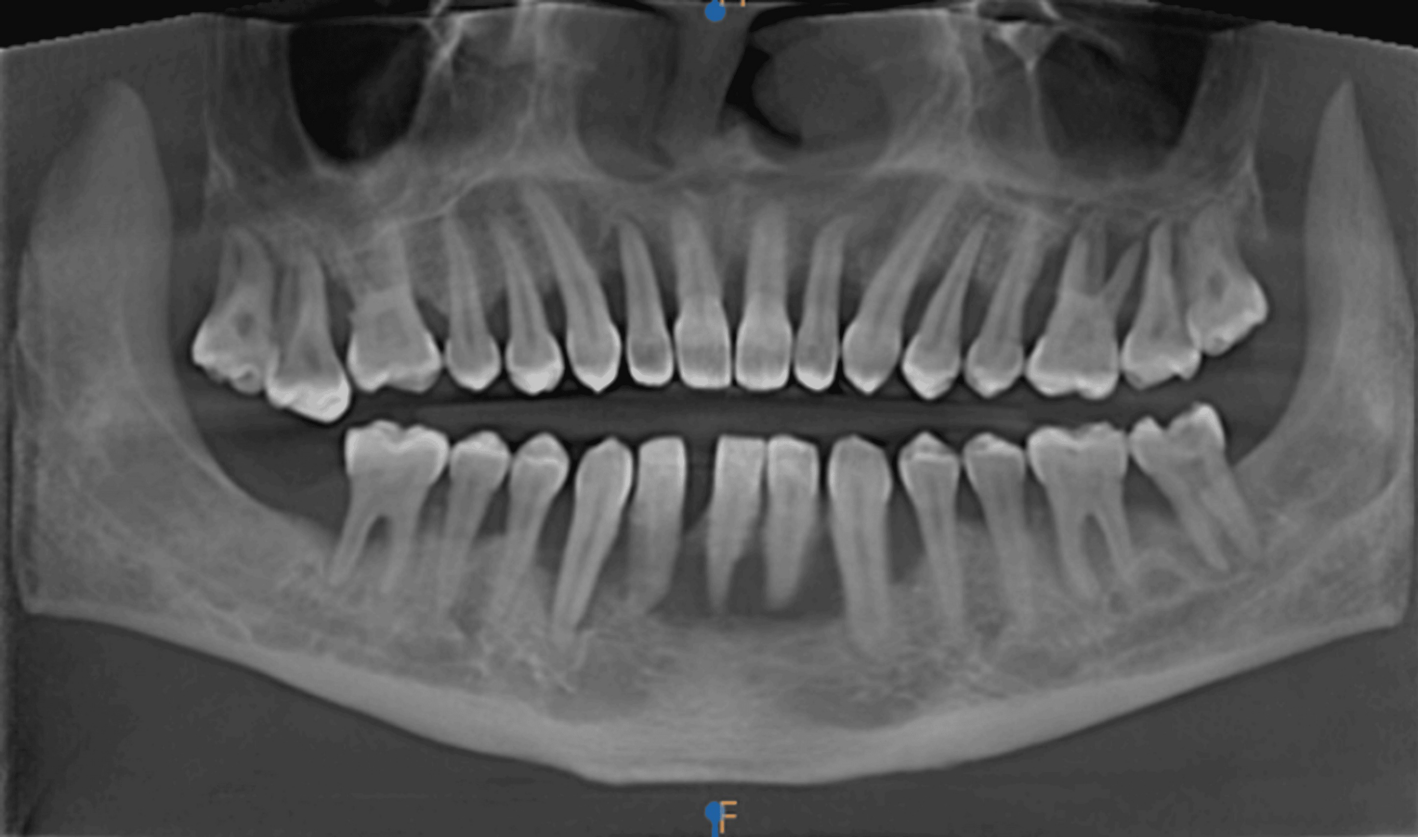
Generalized chronic periodontitis with a breach observed in the left maxillary sinus

To rule out the association between maxillary sinus pathologies and their relation to dental findings, periapical lesions (PLs) are shown in Figures 11-13.

## Results

A total of 300 CBCT scans (comprising 600 maxillary sinuses) were retrospectively analyzed to identify incidental observations in the maxillary sinus. There was a prevalence of 52.05% of cases with incidental maxillary sinus findings. Among these, 53.43% were males and 50.65% were females, with incidental maxillary sinus diseases among the 600 maxillary sinus images shown in Table 2.

**Table 2:** Prevalence of incidental pathologic findings among gender

Prevalence of incidental pathologic	Male		Female	
	N	%	N	%
With pathologic findings	78	53.432	78	50.65
Without pathologic findings	68	46.58	76	49.35

Maxillary sinus pathologies were more common in individuals between 46 and 55 years, i.e., Group 4. In 300 datasets, the prevalence of incidental maxillary sinus findings is 21.33% on the right, 24% on the left, 6.67% in both, and absent in 48% of the cases, as shown in Table 3.

**Table 3:** Total prevalence of incidental pathologic findings tabulated for left and right sinus

Age	Left side		Right side		Both side		Absents	
	N	%	N	%	N	%	N	%
All	72	24	64	21.33	20	6.67	144	48

The most frequent findings in the present study were mucosal thickening in the right and left maxillary sinuses, observed in 40.65% and 46.45% of cases, respectively. Radiopacification was noted in 62.71% of right maxillary sinuses and 23.73% of left maxillary sinuses. Polypoid mucosal thickening was similarly prevalent in both right and left maxillary sinuses, each with a rate of 40.21%, as shown in Table 4.

**Table 4:** Prevalence of incidental findings in the maxillary sinus

	Mucosal thickening		Polypoidal mucosal thickening		Opacification	
	N	%	N	%	N	%
Left side	72	46.45	39	40.21	14	23.73
Right side	63	40.65	39	40.21	37	62.71
Both side	20	12.9	19	19.58	8	13.56

The primary reasons for CBCT scans were dental implant site assessment (30.67%), endodontic procedures (29.00%), orthodontic treatment planning (16.67%), complex tooth extractions or osteotomies (8.67%), TMJ evaluations (4.66%), and trauma not involving the maxilla (4.33%). Consequently, CBCT scans are most commonly recommended for dental implant site assessments and endodontic operations, as shown in Table 5.

**Table 5:** Incidence of subjects indicated for CBCT scans CBCT: Cone-bean computed tomography; TMJ: Temporomandibular joint

Indicated	Numbers	%
Dental implant site assessment	92	30.67
Complex tooth extraction or osteotomy	26	8.67
Orthodontic treatment planning	50	16.67
Trauma without involving maxilla	13	4.33
TMJ evaluation	14	4.66
Endodontic procedures	87	29
Periodontal pathology	18	6

Mucosal thickening was observed in 30% of cases with a periapical abscess, 19.52% with a periapical granuloma, 25% with a periapical cyst, and 51.79% with a breach. Polypoidal mucosal thickening was found in 32.50% of cases with a periapical abscess, 13.79% with a periapical granuloma, 50% with a periapical cyst, and 23.21% with a breach. Opacification was present in 37.50% of cases with a periapical abscess, 20.69% with a periapical granuloma, 25% with a periapical cyst, and 25% with a breach, as shown in Table 6.

**Table 6:** The occurrence of incidental maxillary sinus pathologies related to periapical lesions

	Periapical abscess		Periapical granuloma		Periapical cyst		Breach		χ^2^	p
	N	%	N	%	N	%	N	%	281.44	0.0001
Mucosal thickening	12	30	19	19.52	1	25	29	51.79		
Polypoidal mucosal thickening	13	32.5	4	13.79	2	50	13	23.21		
Opacification	15	37.5	6	20.69	1	25	14	25		
Total	40	100	29	100	4	100	56	100		

## Discussion

In this study, 300 CBCT scans (600 maxillary sinuses) were retrospectively analyzed for incidental findings in the maxillary sinus. There were 53.43% males and 50.65% females with incidental maxillary sinus diseases among the 600 maxillary sinus images. In our sample, 52.05% of cases had incidental maxillary sinus findings. This was slightly higher than in the following studies: Vallo et al. [8] (19%), Munde et al. [9] (44.9%), and Pazera et al. [4] (46.8%). The incidence of incidental maxillary sinus findings was higher in the research listed below than in our investigation: Ritter et al. [7] (56.3%), Hähnel et al. [6] (63%), and Bolger et al. [10] (82.2%). This might be a result of the various indications for CBCT scans used in the studies.

In the present study, maxillary sinus pathologies were more common in individuals between 46 and 55 years (i.e., Group 4), which did not correlate with the following studies. Possible factors include lifestyle, systemic health conditions, and dental practices specific to this age group. In the study by Malik et al. [11], the second decade had a greater incidence, while in the study by Raghav et al. [12], those between the ages of 30 and 39 had a higher prevalence of diseases in the maxillary sinus. Ritter et al. [7] claimed that individuals over 60 years frequently experienced pathologic findings. In 300 datasets, the incidence of incidental maxillary sinus findings was 21.33% on the right, 24% on the left, 6.67% in both, and absent in 48% of the cases. This result follows Oliveira et al. [13], who reported that 46.3% of patients had incidental maxillary sinus findings on the right side and 46.9% on the left side.

The differences in prevalence between the findings of this study and those of other studies and existing literature could be attributed to several factors. First, a diversity of patient groups and ages were examined in various studies. Next, there were differences in sample sizes, applied classification methods, and definitions of pathologic alterations present between the studies [14,15]. In our study, the most common indication for CBCT scans was dental implant site assessment (30.67%). These findings align with Ritter et al.'s study, where 26.53% of CBCT scans were for dental implant site assessment, and with Raghav et al.'s study, which reported 24.3% for dental implant site assessment and 23.1% for endodontic purposes [7,11].

In the present study, mucosal thickening was observed in 30% of cases with a periapical abscess, 19.52% with a periapical granuloma, 25% with a periapical cyst, and 51.79% with a breach. These results are consistent with the findings of systematic reviews by Eggmann et al. [14], Shanbhag et al. [15], and Lu et al. [16]. Polypoidal mucosal thickening was found in 32.50% of cases with a periapical abscess, 13.79% with a periapical granuloma, 50% with a periapical cyst, and 23.21% with a breach. The prevalence reported by Zadsirjan et al. [17] and Nunes et al. [18] ranged from 6.5% to 19.4% and 23%, respectively.

Opacification was observed in 37.50% of cases with a periapical abscess, 20.69% with a periapical granuloma, 25% with a periapical cyst, and 25% with a breach. The size of the PL on radiographs can indicate how the lesion is progressing [19]. Rege et al. reported incidental maxillary sinus findings in 68.2% of the cases in their study [20]. In general, factors including host resistance, infection-causing agents, and anatomical changes in different people - such as the existence and placement of blood and lymphatic arteries - may all affect the propagation of periapical infection to the maxillary sinus.

Since there were two observers in the investigation, inter- or intra-observer reliability could be assessed [21]. However, the present study had a few limitations. First, the sample size was too small to accurately represent the prevalence in the Tamil Nadu population. Sample collection should be significantly increased across various CBCT centers in Tamil Nadu. The lower dose of CBCT imaging compared to CT imaging may be one advantage for imaging the paranasal sinuses. Since this was a retrospective investigation, information about the subjects' smoking behaviors, which may be a factor in male sinus alterations, was not available. Due to their similar radiographic appearance, polyps and retention cysts could not be distinguished from one another. To enhance the precision of patient selection, future studies should incorporate clinical assessments and evaluate the medical data of participants.

Hence, the present study states that dental professionals should have a bird's-eye view when treating chronic odontogenic infections near the maxillary sinus. Early diagnosis and prompt treatment of odontogenic infections help prevent maxillary sinus pathologies. In the future, increasing the sample size and collecting samples from various CBCT centers will yield better results.

Clinical significance

In CBCT, pathologic alterations that are discovered accidentally must frequently be followed and treated. This study adds valuable insights for clinicians on optimizing the use of CBCT in routine dental evaluations, as well as in more complex cases. By highlighting its utility, the research will enhance patient care, improve diagnostic accuracy, and reduce the risk of complications stemming from undetected sinus and dental conditions.

## Conclusions

Using CBCT imaging, the detection of diseases in the maxillary sinus appears to be possible. The most frequently occurring sinus abnormalities were periapical radiolucent lesions associated with the maxillary posterior teeth. Due to the lack of a prior radiographic assessment and patient medical history information, CBCT scans should be interpreted cautiously.
